# Successive complications after anterior cervical fixation: pharyngoesophageal diverticulum, fistulization, and cervical spondylitis by *Streptococcus milleri* – case report and literature review

**DOI:** 10.1186/s13256-019-2037-4

**Published:** 2019-04-30

**Authors:** Patricia Volkow-Fernández, Beda Islas-Muñoz, Patricio Santillán-Doherty, Enrique Estrada-Lobato, Luis Alva-López, José Ávila-Ramírez

**Affiliations:** 1 Infectious Disease Department, Instituto Nacional de Cancerología (INCan), Secretaría de Salud (SSA), San Fernando 22, Col. Sección XVI, Tlalpan, México City, DF 14080 México; 2 Instituto Nacional de Enfermedades Respiratorias, Secretaria de Salud (SSA), México City, Mexico; 3 Nuclear Medicine Department, Instituto Nacional de Cancerología (INCan), Secretaría de Salud (SSA), Mexico City, Mexico; 4 Image Department, Hospital Medica Sur, Mexico City, Mexico; 5 Neurosurgey, Hospital Medica Sur, Mexico City, Mexico

**Keywords:** Pharyngoesophageal diverticulum, Esophageal diverticulum, Anterior cervical spine surgery, Anterior cervical discectomy and fusion complications

## Abstract

**Introduction:**

Pharyngoesophageal diverticulum is an uncommon complication after anterior cervical discectomy and fusion surgery.

**Case presentation:**

Our patient was a 48-year-old woman with two previous cervical surgeries with fixation of C4-C5 and C5-C6, the last one in 2003. Two years after surgery, she presented with arthralgia, arthritis, chills, and fluctuating rash. In 2007, she presented with dysphagia, halitosis, and sputum production. She was diagnosed with a pharyngoesophageal diverticulum with a fistula to C6 vertebra and secondary spondylitis*.* She was taken for open surgery with removal of screws and plates, cricopharyngeal myotomy, and esophageal repair. *Streptococcus milleri* grew in tissue and osteosynthetic material. She received 4 months of amoxicillin and probenecid and had a complete recovery. Since 1991, 19 similar cases have been reported with one fatality. To our knowledge, this is the first reported case of diverticulum complicated with fistula and secondary spondylitis.

**Conclusions:**

In patients with a history of anterior cervical discectomy and fusion complaining of dysphagia, even years after surgery, it is mandatory to perform an esophagogram. This symptom was referred to in 88% of the cases reported in the literature.

## Introduction

Since the 1990s, the number of anterior cervical discectomy and fusion (ACDF) surgeries performed has dramatically increased. The Nationwide Inpatient Sample estimated 932,009 hospital discharges associated with cervical spine surgery from 1992 to 2001 in the United States. The overall rate of complications has been reported to be 3.93%, and mortality was estimated at 0.14%. Factors associated with complications were advanced age, primary diagnosis, and type of surgical procedure. The most common reported complication was dysphagia [1]. This symptom predominates in women and is associated with different types of damage such as vocal cord paralysis, esophageal strictures, or hardware extrusion. Dysphagia caused by pharyngeal or esophageal diverticulum is uncommon. Diverticula are thought to be caused by internal pulsion forces and secondary incoordination between the pharyngeal phase of swallowing and cricopharyngeal relaxation [2]. We present a case of a patient with multiple complications after ACDF that presented a challenge for diagnosis and treatment.

## Case presentation

Our patient was a 48-year-old woman with a history of two previous cervical surgeries, the first one in 1987 and the second in 2003, with placement of titanium plates and screws at C4-C5 and C5-C6. She was seen at the clinic in 2005 with a 2-month history of fatigue, chills, headache, nausea, and asymmetric arthralgia. She also had episodes of malar rash after sun exposure and cutaneous fluctuating rash in the trunk. Physical examination revealed arthritis of the left shoulder and left ankle, livedo reticularis, and erythematous cutaneous rash in the thorax. No infection foci were detected. Laboratory studies revealed thrombocytosis 485,000 cells/mm^3^ (normal range 130,000–400,000 cells/mm^3^), elevated C-reactive protein (CRP) 75 mg/dl (normal range 0.1–1.0 mg/dl), and erythrocyte sedimentation rate (ESR) 40 mm/h (normal range 0–20 mm/h). Autoantibodies were negative, and complement levels were within normal range.

From 2005 to 2007, she had no treatment, and her symptoms had a fluctuating course. In 2007, fatigue, rash, and arthralgia appeared again, and she developed edema in her hands and feet. Rheumatology started prednisone and methotrexate without improvement. Six months later, dysphagia, halitosis, and “sputum” production of purulent aspect were added to the patient’s symptoms. She consulted an ear, nose, and throat specialist, who did not find any abnormality.

**Fig. 1:**
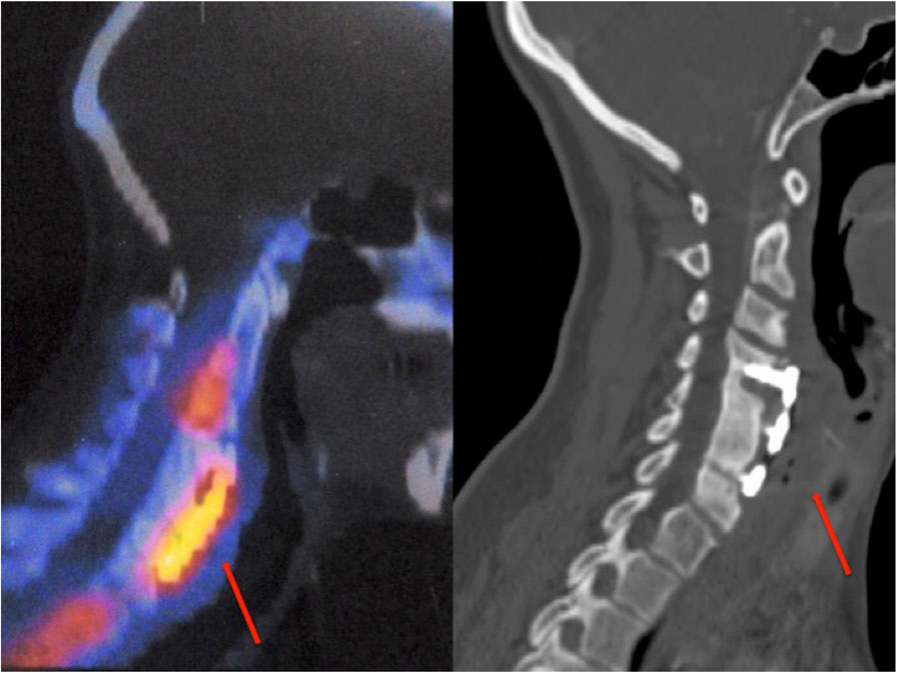
Single-photon emission computed tomography shows spondylitis in the cervical spine (C4-C6) and an inflammatory process in the nasopharynx with free air in subcutaneous tissue and increase in the prevertebral space of > 2 cm (*red arrow*)

**Fig. 2:**
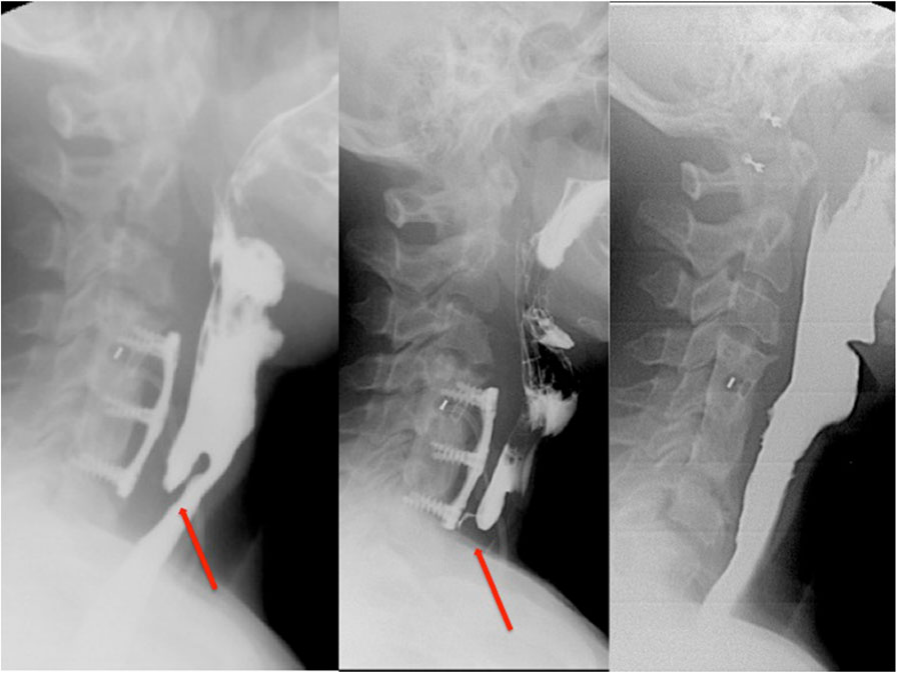
Barium esophagogram shows posterior pharyngoesophageal diverticulum and fistula with leakage from the diverticulum toward C6 vertebra (*red arrows*)

**Fig. 3:**
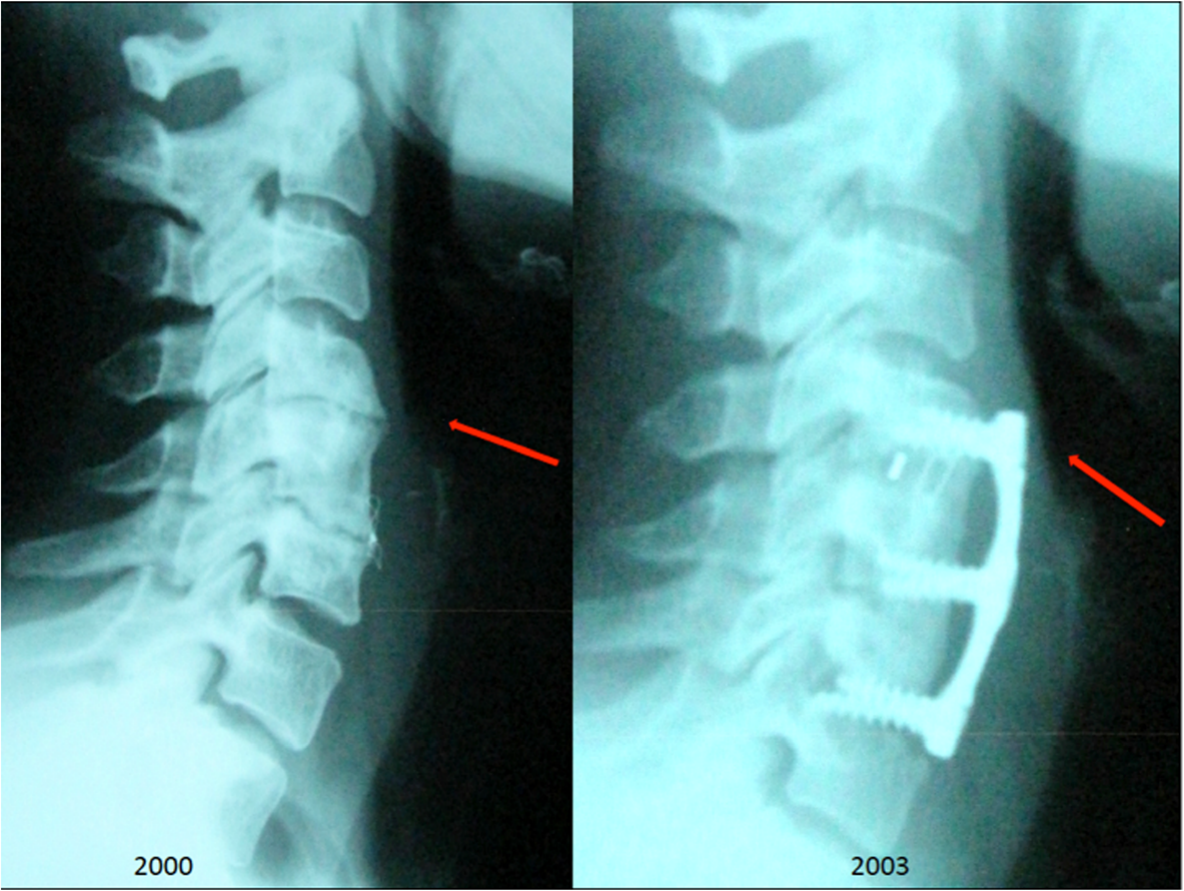
Left: Lateral cervical spine x-ray taken in 2000 showing the fixation material with preserved prevertebral space. Right: Lateral cervical spine x-ray after surgical fixation with intersomatic box to C4-C5 showing preserved prevertebral space and fixation material 5 mm anterior to the vertebral bodies, reducing the retroesophageal space and pressing on the esophagus

She continued with elevated CRP, ESR, and thrombocytosis. Labeled leukocyte single-photon emission computed tomography (SPECT) suggested spondylitis in the cervical spine (C4-C6) and revealed an inflammatory process in the nasopharynx, an increase in the prevertebral space of > 2 cm, and free air in this area (Fig. 1). An esophagogram with hydrosoluble contrast revealed a posterior pharyngoesophageal diverticulum with a fistula to C6 (Fig. 2). The patient’s x-rays of the lateral column after the cervical spine anterior fixation in 2003 showed preserved prevertebral space, and intersomatic C4-C5 box and plate were 5 mm anterior to the vertebral bodies, pressing the esophagus (Fig. 3).

The patient was taken to surgery; screws and plates were removed from C4 to C6; surgical debridement was performed; and the fistula and diverticulum were removed with cricopharyngeal myotomy and esophageal repair. Esophagography with water-soluble contrast showed no leak after surgery, but the lumen of the esophagus at C4–C6 was increased in diameter with diminished compliance. Removed plates, screws, and tissue were cultured and grew *Streptococcus milleri*. The patient was treated with oral amoxicillin 1 g every 8 h and probenecid for 4 months, until a gammagram was negative. Her fatigue, arthralgia, rash, and livedo reticularis as well as dysphagia disappeared. Her acute-phase reactants normalized.

## Discussion

We conducted research in the PubMed database using the following terms: pharyngoesophageal diverticulum, Zenker diverticulum, pharyngeal diverticulum, esophageal diverticulum, anterior cervical spine surgery, anterior cervical discectomy and fusion complications. We obtained 15 articles that were either case reports or case series of diverticulum formation after anterior cervical surgery with a total of 19 cases since 1991 [1–15].

Of the 20 patients, including our patient, 10 were women (50%), and their mean age was 44 years with a range from 24 to 63 years. Seventeen cases (85%) reported dysphagia as the main symptom, and the mean time between the surgery and the symptoms was 4.4 years (interval from 6 months until 18 years); only one case did not specify time [6]. Seventy-seven percent of patients had cervical intervention at levels C5-C6 and/or C6-C7. Eighteen cases (90%) were treated with open surgery, one with endoscopic repair and one with conservative management and surveillance. Two cases were complicated with fistula; one of these was a pharyngocutaneous fistula secondary to neck abscess drainage [2, 4]. Three cases were complicated with abscess or infection: two with neck abscess [2, 6] and one fatal case with perforation, mediastinitis, and death [8]. All of the cases except one had complete resolution of symptoms after surgery. Table 1 lists characteristics of the different cases, including general information of the patients, time elapsed from surgery until symptoms appeared, level of cervical intervention, location of diverticulum, procedure performed, and outcome [1–15]. Esophageal perforation following ACDF has an incidence of 0.2–1.15%; some cases have been reported without a finding of fistula or diverticulum in which the diagnosis may have been made belatedly [16–20].

**Table 1:** Characteristics of cases reported with diverticulum as complication of cervical anterior fixation

No.	Author, year [reference]	Sex	Age (yr)	Time elapsed after surgery	Clinical manifestations	Location of cervical intervention and diverticulum	Procedure	Outcome
1	Goffart *et al*., 1991 [3]	M	44	11 mo	Dysphagia and regurgitation	C6-C7 Hypopharyngeal	Open repair with cricopharyngeal myotomy	Recovered
2	Salam *et al.*, 1994 [4]	F	36	2 yr	Dysphagia	Not specified Posterior pharyngeal	Endoscopic repair	Recovered
3	Sood *et al.*, 1998 [5]	M	45	13 yr	Odynophagia and dysphagia	C5-C7 Posterior pharyngeal	Open repair and cricopharyngeal myotomy	Recovered
4	Ba *et al*., 2006 [6]	F	28	NS	Odynophagia, dysphagia, and regurgitation	C5-C6 Posterior pharyngeal	Open repair of the pharyngeal diverticulum with myotomy Complicated with postoperative neck abscess	Recovered
5	Ba *et al.*, 2006 [6]	M	63	6 mo	Dysphagia, globus sensation, and regurgitation	NS Posterior esophageal	Open repair and cricopharyngeal myotomy	Recovered
6	Summers *et al*., 2007 [7]	F	43	2 yr	Odynophagia, fever, weight loss, and neck pain	C4-C7 Pharyngoesophageal	Complicated with retropharyngeal abscess, perforation, and mediastinitis	Death
7	Joanes *et al.*, 2008 [8]	M	31	3 yr	Mild dysphagia	C5-C7 Pharyngoesophageal	Open repair, removal of plate and screws, and esophageal reconstruction	Recovered
8	Reboll Ferrer *et al*., 2008 [13]	M	35	2 yr	Progressive dysphagia, globus sensation, and regurgitation	C5-C7 Pharyngoesophageal	Open repair with diverticulectomy by left lateral cervicotomy	Recovered
9	Solerio *et al*., 2008 [15]	M	41	7 yr	Fever, dysphagia, and fistulous orifice	C4-C6	Open repair with sternocleidomastoid muscle flap interposed between the pharyngoesophageal junction and the cervical spine	Recovered
10	Alexander *et al*., 2008 [14]	F	50	6 yr	Dysphagia, regurgitation, halitosis	C5-C7 Hypopharyngeal	Open repair, removal of the hardware, followed of endoscopic staple diverticulotomy	Recovered
11	Allis *et al*., 2010 [2]	F	56	1 yr	Regurgitation and choking spells	C5-C6 Hypopharyngeal	Open repair, removal of hardware, resection of the diverticulum, pharyngeal repair, and cricopharyngeal myotomy	Recovered
12	Allis *et al*., 2010 [2]	F	59	2 yr	Progressive dysphagia and regurgitation	C4-C6 Hypopharyngeal	Open repair, diverticulectomy with cricopharyngeal myotomy	Recovered
13	Allis *et al*., 2010 [2]	F	50	1 yr	Left neck abscess	C5-C7 Hypopharyngeal with neck abscess and pharyngocutaneous fistula	Abscess drainage with conservative management of the diverticulum because size was less than 1 cm Surveillance	Recovered
14	Tian *et al*., 2011 [9]	M	31	7 yr	Dysphagia, odynophagia, fever, and weight loss	C4-C5 Pharyngoesophageal	Open repair, removal of plate and screws, and esophageal reconstruction	Recovered
15	Anandaswamy *et al*., 2012 [1]	M	59	2 yr	Dysphagia, tingling, and weakness in both the upper limbs	C5-C6	Posterior pharyngoesophageal perforation with extrusion of the plate and screw Open repair with removal of cervical implants	Recovered
16	Almre *et al*., 2014 [10]	M	53	18 yr	Progressive dysphagia	C6-C7 Pharyngoesophageal	Open repair, removal of plate and screws, and cricopharyngeal myotomy	Recovered
17	Sadrizadeh *et al*., 2015 [11]	F	46	7 yr	Dysphagia, halitosis, and chest pain	Not specified Posterior esophageal pseudodiverticulum	Open excision of diverticulum without esophageal repair because of severe fibrosis	Recovered
18	Sadrizadeh *et al*., 2015 [11]	M	24	2.5 yr	Odynophagia and dysphagia	C5-C6 Posterior pharyngeal pseudodiverticulum	Open repair and cricopharyngeal myotomy	Recovered
19	Park *et al*., 2016 [12]	F	54	3 yr	Dysphagia and sense of irritation in the neck	C5-C7 Posterior pharyngeal	Open repair with cricopharyngeal myotomy	Recovered
20	Our patient	F	48	4 yr	Dysphagia, halitosis, fever, and immune complex disease	C4-C6 Pharyngoesophageal	Diverticulum fistulization with secondary C6 infectious spondylitis Cricopharyngeal myotomy and open esophageal repair + 4 mo amoxicillin	Recovered

*NS* Not specified

In our patient, cervical spondylitis developed due to fistulization at C6, allowing for contiguous spread of oral flora and infection toward the cervical spine and soft tissues. Chronic infection caused an immune complex disease, which resolved after removal of fixation plates and screws, surgical repair of the fistula and diverticulum, and prolonged antimicrobial therapy. The causative agent is part of the normal oral flora. Our hypothesis is that the esophageal diverticulum developed after the anterior cervical spine fixation performed in 2003, because the fixation plate’s position was too anterior (Fig. 3), pressing on the esophagus during deglutition, which caused erosion and fistula formation. This case shows multiple complications secondary to anterior cervical fixation. To our knowledge, this is the first reported case of a patient with fistulization to the cervical spine and secondary infectious spondylitis caused by pharyngoesophageal diverticulum secondary to ACDF.

## Conclusions

This case exemplifies how new therapeutic strategies cause problems not previously described and that meticulous clinical evaluation and rational use of diagnostic workup are necessary to solve clinical issues. Persistent dysphagia following anterior cervical surgery should alert the clinician to the possibility of having different possible complications and is mandatory for a complete diagnostic approach to rule out this possibility.

## Abbreviations

ACDF: Anterior cervical discectomy and fusion
C4, C5, C6: Cervical vertebrae 4, 5, and 6
CRP: C-reactive protein
ESR: Erythrocyte sedimentation rate
SPECT: Single-photon emission computed tomography

## References

[1] Anandaswamy TC, Pujari VS, Shivanna S, Manjunath A. Delayed pharyngoesophageal perforation following anterior cervical spine surgery: an incidental finding. J Anaesthesiol Clin Pharmacol. 2012;28(1):139–40. 10.4103/0970-9185.92477. PMC3275960 22345975

[2] Allis TJ, Grant NN, Davidson BJ. Hypopharyngeal diverticulum formation following anterior discectomy and fusion: case series. Ear Nose Thorax J. 2010;89(11):E4–9. 10.1177/014556131008901102 21086270

[3] Goffart Y, Lenelle J, Moreau P, *et al.* Traction diverticulum of the hypopharynx following anterior cervical spine surgery. Ann Otol Rhinol Laryngol. 1991;100:852–5. 10.1177/000348949110001012. 1952654

[4] Salam MA, Cable HR. Acquired pharyngeal diverticulum following anterior cervical fusion operation. Br J Clin Pract. 1994;48:109–10. 8024986

[5] Sood S, Henein RR, Girgis B. Pharyngeal pouch following anterior cervical fusion. J Laryngol Otol. 1998;112:1085–6. 10.1017/s0022215100142525 10197151

[6] Ba AM, LoTempio MM, Wang MB. Pharyngeal diverticulum as a sequela of anterior cervical fusion. Am J Otolaryngol. 2006;27(4):295–7. 10.1016/j.amjoto.2005.11.009. 16798413

[7] Summers LE, Gump WC, Tayag EC, Richardson DE. Zenker diverticulum: a rare complication after anterior cervical fusion. J Spinal Disord Tech. 2007;20(2):172–5. 10.1097/BSD.0b013e31802c1474 17414989

[8] Joanes V, Belinchon J. Pharyngoesophageal diverticulum following cervical corpectomy and plating. Case report. J Neurosurg Spine. 2008;9:258–60. 10.3171/SPI/2008/9/9/258. 18928221

[9] Tian H, Yuan W, Johnson JS, Chen H, Chen D. Pharyngoesophageal diverticulum: a delayed complication of anterior cervical spine surgery. Eur. Spine J. 2011;20(Suppl 2):S211–6. 10.1007/s00586-010-1579-3 PMC3111524 20927556

[10] Almre I, Asser A, Laisaar T. Pharyngoesophageal diverticulum perforation 18 years after anterior cervical fixation. Interact Cardiovasc Thorac Surg. 2014;18(2):240–1. 10.1093/icvts/ivt421 PMC3895056 24246672

[11] Sadrizadeh A, Soltani E, Abili M, Dehghanian P. Delayed esophageal pseudodiverticulum after anterior cervical spine fixation: report of 2 cases. Iran J Otorhinolaryngol. 2015;27(79):155–8. PMC4409960 25938087

[12] Park J, Chang W, Do Hyung K. Acquired pharyngeal diverticulum after anterior cervical fusion operation misdiagnosed as typical Zenker diverticulum. Korean J Thorac Cardiovasc Surg. 2016;49:309–12. 10.5090/kjtcs.2016.49.4.309. PMC4981237 27525244

[13] Reboll Ferrer RM, García Navalón C, Armengot Carceller M, Basterra Alegría J. Post-traumatic iatrogenic pharyngoesophageal diverticulum. Acta Otorrinolaringol Esp. 2008;59(10):510. 19080791

[14] Alexander RE, Silber J, Myssiorek D. Staged surgical management of hypopharyngeal traction diverticulum. Ann Otol Rhinol Laryngol. 2008;117(10):731–3. 10.1177/000348940811701004. 18998499

[15] Solerio D, Ruffini E, Gargiulo G, *et al.* Successful surgical management of a delayed pharyngo-esophageal perforation after anterior cervical spine plating. Eur Spine J. 2008;17 Suppl 2:S280–4. 10.1007/s00586-007-0578-5. PMC2525898 18224356

[16] Wang MC, Chan L, Maiman DJ, *et al.* Complications and mortality associated with cervical spine surgery for degenerative disease in the United States. Spine. 2007;32(3):342–7. 10.1097/01.brs.0000254120.25411.ae. 17268266

[17] Cheung JPY, Luk KDK. Complications of anterior and posterior cervical spine surgery. Asian Spine J. 2016;10(2):385–400. 10.4184/asj.2016.10.2.385. PMC4843080 27114784

[18] Lu X, Guo Q, Ni B. Esophagus perforation complicating anterior cervical spine surgery. Eur Spine J. 2012;21(1):172–7. 10.1007/s00586-011-1982-4. PMC3252451 21874293

[19] Hershman SH, Kunkle WA, Kelly MP, *et al.* Esophageal perforation following anterior cervical spine surgery: case report and review of the literature. Global Spine J. 2017;7(1 Suppl):28S–36S. 10.1177/2192568216687535. PMC5400185 28451488

[20] Patel NP, Wolcott WP, Johnson JP, Cambron H, Lewin M, McBride D, Batzdorf U. Esophageal injury associated with anterior cervical spine surgery. Surg Neurol. 2008;69(1):20–4. 10.1016/j.surneu.2007.05.006 17976697

